# Draft Genome Sequence of Rhodococcus erythropolis VKPM Ac-1659, a Putative Oil-Degrading Strain Isolated from Polluted Soil in Siberia

**DOI:** 10.1128/MRA.00535-21

**Published:** 2021-07-22

**Authors:** A. A. Korzhenkov, E. D. Bakhmutova, A. O. Izotova, A. A. Bavtushnyi, K. V. Sidoruk, E. V. Patrusheva, M. V. Patrushev, S. V. Toshchakov

**Affiliations:** a National Research Center “Kurchatov Institute,” Moscow, Russia; b National Research Center “Kurchatov Institute”—GOSNIIGENETIKA, Moscow, Russia; University of Southern California

## Abstract

The draft genome sequence of Rhodococcus erythropolis VKPM Ac-1659, a putative oil-degrading strain, is reported. This genome sequence may provide better insights into the diversity and evolution of the genes responsible for hydrocarbon degradation in soil microorganisms.

## ANNOUNCEMENT

Representatives of the species Rhodococcus erythropolis, first described by Gray and Thornton in 1928 as aromatic compound decomposers (1), are widely used in bioremediation technologies as bioemulsifiers and hydrocarbon degraders (2). Here, we report the draft genome sequence of R. erythropolis VKPM Ac-1659, a putative oil-degrading strain isolated from oil-polluted soil collected about 753 km from the city of Novosibirsk, Russia, and deposited in the Russian National Collection of Industrial Microorganisms (VKPM; https://vkpm.genetika.ru/).

For DNA extraction, the strain was grown on meat-peptone medium (3) at 29°C for 24 h. DNA was isolated using the ammonium salt treatment technique, as described previously (4). A genomic library was prepared using the KAPA HyperPlus kit (Roche, Switzerland) according to the manufacturer’s recommendations. Sequencing was performed on an Illumina MiSeq system using a 2 × 250-bp format and a MiSeq reagent kit; 1,745,674 read pairs were obtained, yielding 866.4 million nucleotides.

Default parameters were used for all software unless otherwise specified. Quality control of the raw reads was performed using the fastp v0.20.1 package (5). Read processing and genome assembly were conducted using the ZGA pipeline (6), with the following steps: (i) quality read trimming and adapter removal using BBDuk v38.75, (ii) merging of the overlapping paired reads using bbmerge v38.75 (7), and (iii) *de novo* assembly of the assembled treated reads using the SPAdes v3.13.1 assembler (8). The genome assembly consisted of 170 contigs with a total length of 6,612,324 bp and an *N*_50_ value of 664,755 bp. The final genome coverage was 74×. The G+C content of the assembly was 62.38%. Analysis of the assembly quality using CheckM (9) showed both a high level of completeness (99.94%) and a low level of predicted contamination (3.24%). Genome-based taxonomic classification of the sequenced strains was performed using the Type Strain Genome Server (TYGS) (10). The TYGS analysis identified strain VKPM Ac-1659 as Rhodococcus erythropolis with high confidence; the estimated digital DNA-DNA hybridization (dDDH) values were greater than 85% for both the R. erythropolis type strains, NBRC 15567 and JCM 3201. Genome annotation was performed during submission of the data to NCBI using the NCBI Prokaryotic Genome Annotation Pipeline (11).

Analysis of the genes responsible for oil degradation was carried out using NCBI BLAST+ (12) with known oil degradation genes of both marine (13) and soil (14) microorganisms as queries. Two gene clusters were detected, including genes for rubredoxins, alkane 1-monooxygenase, and TetR family transcriptional regulators, the last of which might be responsible for the first step of alkane utilization, as described previously (15). One cluster consisted of AC1659_RS04735, AC1659_RS04740, AC1659_RS04745, and AC1659_RS04750, and the other consisted of AC1659_RS17110 AC1659_RS17115, AC1659_RS17120, and AC1659_RS17130. An additional contribution to alkane metabolism is possibly made by two NAD(P)/FAD-dependent oxidoreductases (AC1659_RS20515 and AC1659_RS22365) showing a high level of similarity with their counterparts in Acinetobacter baylyi ADP1, known to metabolize a wide spectrum of substrates, including phenol, benzyl alcohol, benzaldehyde, benzoate, and their hydroxy derivatives (16). In turn, the genes for polycyclic aromatic hydrocarbon degradation were not identified.

### Data availability.

The genome sequence has been deposited at NCBI GenBank and is available under accession number JAGVVT000000000.1. The raw sequencing reads are available in NCBI SRA under accession number SRX10910539.
